# FIAEs in Famous Faces are Mediated by Type of Processing

**DOI:** 10.3389/fpsyg.2012.00256

**Published:** 2012-08-01

**Authors:** Peter J. Hills, Michael B. Lewis

**Affiliations:** ^1^Department of Psychology, Anglia Ruskin UniversityCambridge, UK; ^2^School of Psychology, Cardiff UniversityCardiff, UK

**Keywords:** adaptation, aftereffects, face processing, familiar faces, unfamiliar faces

## Abstract

An important question regarding face aftereffects is whether it is based on face-specific or lower-level mechanisms. One method for addressing this is to explore how adaptation in upright or inverted, photographic positive or negative faces transfers to test stimuli that are either upright or inverted and normal or negated. A series of studies are reported in which this is tested using a typical face identity aftereffect paradigm in unfamiliar and famous faces. Results showed that aftereffects were strongest when the adaptor matched the test stimuli. In addition, aftereffects did not transfer from upright adaptors to inverted test images, but did transfer from inverted adaptors to upright test images in famous faces. However, in unfamiliar faces, a different pattern was observed. The results are interpreted in terms of how identity adaptation interacts with low-level adaptation and highlight differences in the representation of famous and unfamiliar faces.

## Introduction

Face distortion aftereffects (FDAEs) have been reported whereby adaptation to a face distorted in one direction (e.g., compressed) will cause post-adaptation faces to appear distorted in the opposite direction (e.g., expanded; Webster and MacLin, 1999; Yamashita et al., 2005; Carbon et al., 2007; Little et al., 2008). One critical question is whether aftereffects in face recognition reflect expert face-specific mechanisms or lower-level generalized mechanisms (Hole, 2011). Adaptation is typically said to be due to some sort of fatigue of cells that respond to a particular characteristic (e.g., Ferster and Miller, 2000). Low-level adaptation is tied closely to the physical properties of the stimuli: the adaptor must match the test stimuli. For higher-level adaptation mechanisms, the adaptor and the test do not have to match so well and aftereffects can transfer across viewpoints and images. There is sufficient evidence to suggest that both lower- and higher-level adaptation mechanisms are involved in face aftereffects, but the relative involvement of each is not well understood.

In their seminal study, Webster and MacLin (1999) created a series of stimuli of faces that were distorted from the norm in a Gaussian fashion in vector format. The resulting set of faces was presented to participants using a nulling-match procedure, whereby participants had to adjust a distorted face to appear normal. After inspecting an adaptation face for 5 min and for 8 s between each test image, participants had to adjust the distorted face such that it would appear normal. The adjustments the participants made were distorted in the opposite direction to the adaptation stimuli the participants had seen. The results were replicated in a normal rating procedure.

Webster and MacLin also noted that adaptation to an undistorted face was not possible: in other words, staring at a normal face did not affect the perception of distorted faces. Moreover, aftereffects transferred across faces and even to the perceivers’ own faces. The aftereffects occurred for upright faces and for inverted faces, but only if the orientation of the adaptor face was matched with the orientation of the test faces. The FDAE is partially size-tolerant since it transfers from an adaptor of one size to test stimuli of a different size, even the size difference is a factor of 4 (Zhao and Chubb, 2001). The magnitude of such aftereffects is significantly smaller when the test face and the adaptor do not match. FDAEs also transfer across parts of the retina (Hurlbert, 2001; Anderson and Wilson, 2005) and partially across viewpoints (Jiang et al., 2006). These results indicate that these aftereffects involve at least some higher-level mechanisms.

Yamashita et al. (2005) found that the magnitude of face aftereffects are dependent on the visual similarities between the adaptor and the test stimuli. Nevertheless, changes that affect the recognizability of faces affect the magnitude of aftereffects more than changes that do not affect the recognizability. Size and color differences between the adaptor and the test stimuli reduce the magnitude of adaptation significantly less than spatial frequency and contrast differences between the adaptation and test stimuli.

Often considered similar to FDAEs are face identity aftereffects (FIAEs), whereby the perceived identity of a face is altered after adaptation to a particular identity. Leopold et al. (2001) conducted an elaborate study into FIAEs. In their study, 200 faces were morphed together to produce a prototype face. This was assumed to be the center of the face-space (see Valentine, 1991). Due to the morphing process, each face identity could be measured in terms of Euclidean distances from the prototype face. Thus, a series of faces were created ranging from the prototype face to the face identity, each differing in identity “strength.” Identification thresholds (the required identity strength to perceive the face identity) were taken before and after adaptation to an anti-face identity (opposite from the face identity in terms of Euclidean geometry). Post-adaptation to the anti-face, the identification threshold was lowered by 12.5% suggesting it was easier to perceive the identity following adaptation since the prototypical face is shifted. The magnitude of the aftereffects were similar for upright and inverted faces, provided that the adaptation and test faces were in the same orientation.

Another facet of the FIAE is that it transfers across viewpoints at least in some participants (Jiang et al., 2007, 2009). Their participants were adapted to a face image in one pose and tested on images in the same or different poses. Their results indicated that although significant adaptation occurred when the faces are in a different pose, the magnitude is significantly less than when the images are in the same pose. This study certainly indicates that this adaptation is not solely based on the visual similarity between adaptation and test (and thus higher-level). Similar results were obtained by Benton et al. (2006) in that some of their participants showed adaptation transferring across viewpoints while others did not. Hills et al. (2008) found an individual difference variable that moderated the magnitude of face aftereffects: the ability to visualize whereby participants who were better able to mentally visualize a scene showed larger aftereffects than participants less able to visualize. Suggesting that there is some higher-level mechanism behind these aftereffects leads on to the question of whether this mechanism is face-specific or if is based on shape-aftereffects (e.g., Suzuki, 2001, 2003).

Face recognition is characterized by an expert processing mechanism that relies on the configuration of two eyes above a nose above a mouth rather than processing features independently (typically referred to as configural coding as opposed to featural coding (e.g., Maurer et al., 2002). This configuration is disrupted in an inverted face (Yin, 1969), making it harder to recognize (e.g., Diamond and Carey, 1986). Photographic negation (reversed contrast polarity) of a facial image causes it to be recognized less accurately, but does not alter the type of processing engaged (e.g., Galper, 1970). Photographic negatives are generally associated with more error in encoding rather than a change in processing (e.g., Valentine, 1991; George et al., 1999; Russell et al., 2006).

Few studies have looked at the effect type of processing (expert and potentially face-specific configural coding versus inexpert and object-based featural coding) has on the magnitude of the FIAE. In the FDAE, Watson and Clifford (2003, 2006) have shown that aftereffects do not transfer as readily across orientations. However, aftereffects are observed in inverted faces even when the adaptor is upright, suggesting that adaptation does transfer from expert face-processing mechanisms to inexpert mechanisms. Hole (2011) has shown that adaptation to upright, inverted, or stretched famous faces caused significant aftereffects in upright test faces. This suggests that the FIAE does transfer from inexpertly coded faces to expertly coded ones.^1^

There is one caveat with much of the research presented thus far. It has been conducted on unfamiliar faces. Unfamiliar face perception is based on different mechanisms and neural systems than familiar (personally familiar, experimentally manipulated familiar, famous, or own faces) face perception (Ellis et al., 1979; Tong and Nakayama, 1999; Megreya and Burton, 2006; Gobbini and Haxby, 2007). The representation of familiar faces must be invariant to changes in viewpoint, expression, and other visual changes. This allows them to be recognized from minimal visual information and even from low quality video images (Burton et al., 1999). Unfamiliar faces are difficult to recognize even under optimal conditions (Kemp et al., 1997) because they are represented in a more pictorial and two-dimensional manner (e.g., Ryu and Chaudhuri, 2006). The representations of faces of different levels or types of familiarity is likely to be based on different mechanisms again (e.g., Taylor et al., 2009). Given that the representations of faces depends on levels of familiarity it is important to explore how face aftereffects represent themselves faces that are not unfamiliar. There have been limited studies conducted on adaptation in famous faces specifically.

Jiang et al. (2007) manipulated the level of familiarity participants had with computer-generated faces. In the highest level of familiarity, in which participants were presented with the same face in multiple views for 32 5-s exposures, the aftereffects transferred across viewpoint more so than in the lowest level of familiarity, in which participants were presented with the face in one view only. Furthermore, in the highest level of familiarity, the aftereffects transferred to faces under novel illumination conditions (Jiang et al., 2009). However, the aftereffects demonstrated by Jiang et al. are still in originally unfamiliar faces. Familiar faces have been viewed much more extensively in a variety of contexts and illumination conditions.

Carbon and Leder (2005, 2006) have shown that both the FDAE and the FIAE are longer lasting in famous faces than unfamiliar faces, but do not transfer to other faces in the same way that aftereffects in unfamiliar faces do (Carbon et al., 2007). Hills et al. (2010) have shown that non-visual adaptors can cause aftereffects in famous faces. Prolonged imagination, exposure to the name or to the voice cause aftereffects in faces to a similar degree as adaptation to a different image of the face.

This background summarizes three key areas of face aftereffects that require further elaboration: firstly, whether there is reliance on specific face-processing mechanisms in the FIAE. This can be tested by exploring how the aftereffects transfer from upright to inverted stimuli and vice versa. Secondly, how much (relatively) of the FIAE is low-level and how much is high-level. Part of this can be explored by assessing how aftereffects transfer across different image manipulations and most importantly to different images. A third question is whether the aftereffects are different across famous and unfamiliar faces.

## Experiment 1A

An experiment was conducted that aimed to examine how the FIAE is affected by configural processing. Eight different adaptation stimuli were used comparing the effects of same and different adaptor image from that used at test, whilst also comparing the effects of orientation and negation of the magnitude of adaptation. Two hypotheses can be made regarding this study. Image-based adaptation may occur, whereby adaptation will be greater when the adaptor and test stimuli are matched, regardless of what the adaptor is. However, if the FIAE is based on some form of face-specific coding mechanism, then it is likely to be observed for upright rather than inverted faces.^2^ The difference between Experiments 1a and 1b is that the faces in Experiment 1a were famous, whereas the faces in Experiment 1b were unfamiliar.

## Method

### Participants

Thirty-two (9 male, mean age 21 years) Cardiff University students undertook this experiment as partial fulfillment of a course requirement. All participants had normal or corrected-to-normal vision. All were White British nationals who were familiar with the famous faces.

### Materials

Two different images of George Bush and Tony Blair were collected. They were matched for dimensions (100 mm × 160 mm) and resolution (72 dpi). Image one of George Bush was matched for pose and lighting with image one of Tony Blair. A series of morphs were created using Smartmorph™ Software with 200 anchor points. Fifty morphs were created that ranged from 100% George Bush to 100% Tony Blair in increments of 2% (thus 50 images). Image two of George Bush was in a different pose and under different lighting conditions from image one of George Bush and matched to image two of Tony Blair. The “image two” pair were morphed together in the same way as the image one pair. The 100% images for each identity and each pair were also used as the adaptor.

**Figure 1:**
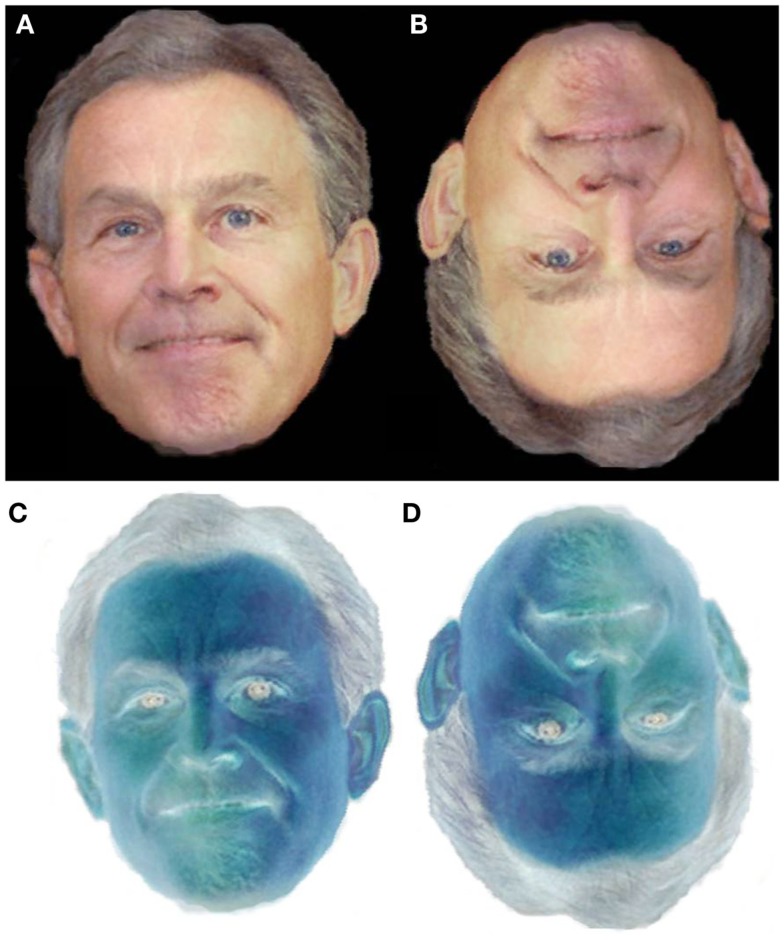
**Examples of the stimuli used in this Experiment**. **(A)** The unaltered 50% midpoint. **(B)** The inverted 50% midpoint. **(C)** The negated 50% midpoint. **(D)** The inverted and negated 50% midpoint.

These two sets of morphs were inverted into two addition sets. Two negated sets were also created using Adobe Photoshop™ image manipulation software. These negated sets were subsequently inverted to create two additional sets of stimuli. The 50% image of each type of stimulus is presented in Figure 1. All stimuli were presented using SuperlabPro 2™ Research Software on an RM PC.

### Design

The adaptor was manipulated between subjects with four levels (same image, different image, negated image, or inverted image). A within-subjects manipulation was also implemented, whereby participants saw eight types of test faces: 2 (same or different image) × 2 (inverted or upright) × 2 (negated or control). The magnitude of adaptation was measured as the change in the PSE pre- to post-adaptation. Participants were randomly allocated to one of the between subjects conditions with the proviso that there was an equal number of participants in each condition (*N* = 12).

### Procedure

Participants were introduced to pictures of George Bush and Tony Blair that they would see in the experiment. The Experiment had three consecutive phases: baseline, adaptation, and test. The baseline phase involved the participants seeing all the morphs 10 times in a random order. They had to make a decision based on whether they thought the image looked more like George Bush (by pressing the G key) or Tony Blair (by pressing T key) based on the methodologies in Levitt (1971). Each morph was on screen until the participant responded. Between each morph a 100-ms Gaussian noise mask was on screen. The purpose of this baseline phase was to discover each individual participant’s “natural” PSE.

Once the baseline had finished, the participants were instructed to rest for 2 min and then given a 3-min irrelevant distractor task. This distractor task involved a participants filling out a questionnaire about their experiences at University. Following this, participants were presented with the adaptor image for 60 s. They were told to examine the image that was presented on screen, which was either George Bush or Tony Blair.

**Figure 2:**
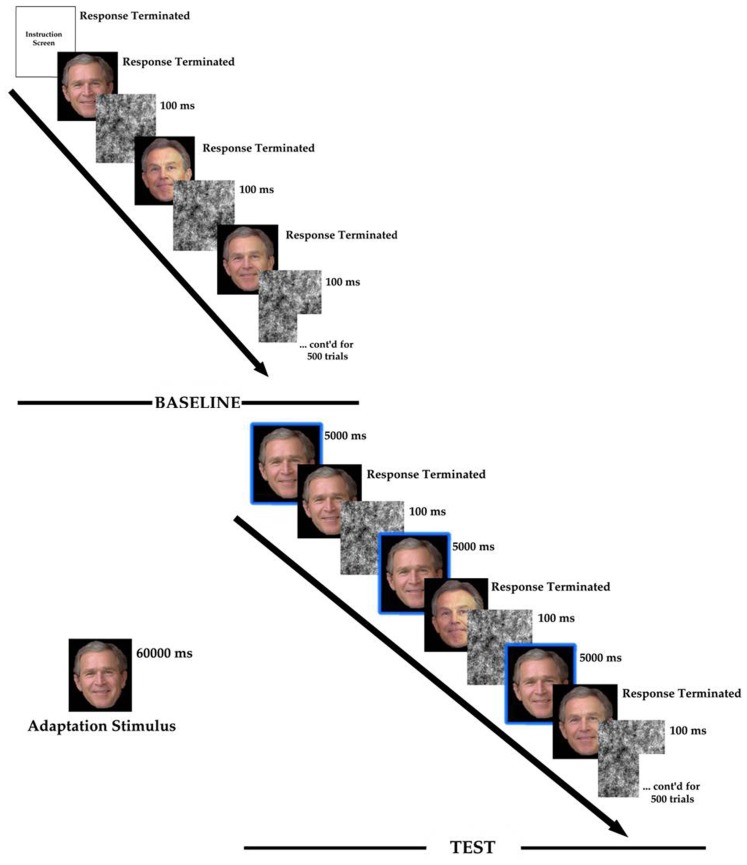
**The procedure for the experimentation**. Those surrounded by the box are the adaptor repeated during the test phase.

Immediately following the adaptor, a repeat of the baseline procedure took place. However, preceding each test face, participants were presented with the adaptor for another 5 s (e.g., Hills et al., 2010). Once the test phase had been completed, participants were thanked and debriefed fully. The total experimentation time for each participant was approximately 75 min. A schematic representation of the procedure is presented in Figure 2.

## Results

**Figure 3:**
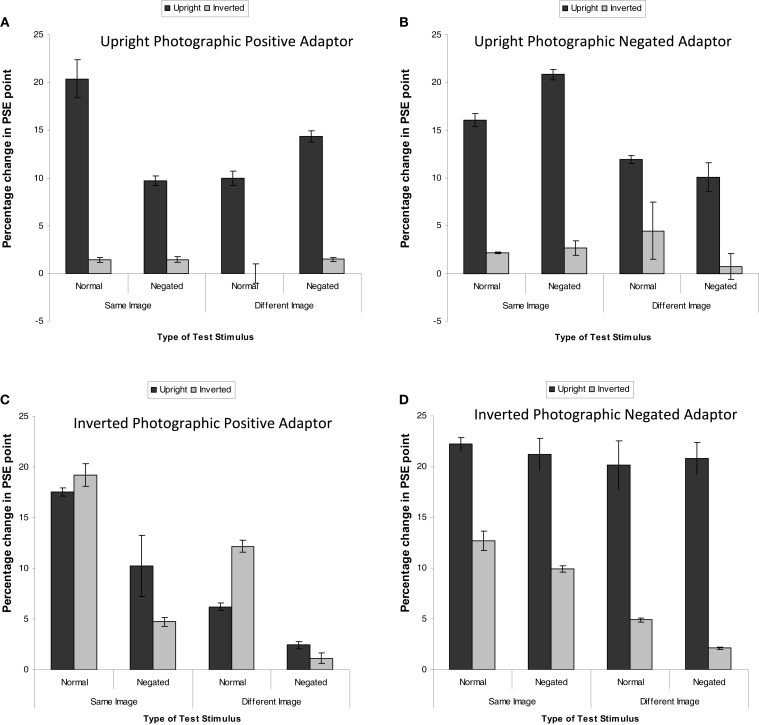
**Mean PSE (in identity strength needed to perceive the adapted identity) shift pre- to post-adaptation (as a measure of the magnitude of the aftereffect) for famous faces, when the adaptor is (A) upright and photographically positive, (B) upright and negated, (C) inverted and positive, and (D) inverted and negated**. Darker bars represent upright test stimuli, lighter bars represent inverted test stimuli. Error bars represent standard error.

The magnitude of adaptation was calculated by subtracting the PSE pre-adaptation from the PSE post-adaptation. There was no effect of image identity or pair, as such the data were collapsed across these variables. Figure 3 shows the mean percentage increase in PSE in the George Busy–Tony Blair continuum for each of the test stimuli for each of the adaptor type. A positive number indicates more identity is needed to perceive the identity of the adaptor, i.e., reduced identity strength. The first analysis was a 4 (adaptor type) × 2 (orientation of test stimuli) × 2 (photographic positive/negative test stimuli) × 2 (same or different image). This revealed a significant four-way interaction, *F*(3, 28) = 16.51, MSE = 3.07, *p* < 0.05, $\eta_{p}^{2}=0.64.$ The four-way interaction is interpreted as the three-way interaction (between orientation, photographic negation, and image-change) is different depending on the adaptor type. This indicates that different adaptor types affect different mechanisms. To explore this, each three-way interaction for each adaptor type was analyzed. In addition, there was also a main effect of adaptor type, *F*(3, 28) = 62.00, MSE = 9.47, *p* < 0.05, $\eta_{p}^{2}=0.87,$ in which aftereffects were larger following adaptation to the negated and inverted stimuli than all other stimuli (all *p*s < 0.05).

### Upright photographic positive adaptor

Figure 3A indicates that greater adaptation occurred when the test stimuli were upright. The data were subjected to a 2 × 2 × 2 within-subjects ANOVA. This revealed that greater adaptation occurred when the same image was used for both adaptation and test, *F*(1, 7) = 66.44, MSE = 0.77, *p* < 0.05, $\eta_{p}^{2}=0.91.$ Greater adaptation was observed for upright test stimuli than inverted test stimuli, *F*(1, 7) = 1664.92, MSE = 1.51, *p* < 0.05, $\eta_{p}^{2}=0.97.$ There were no significant differences in the magnitude of adaptation for negated test stimuli, *F*(1, 7) = 3.44, MSE = 6.46, *p* > 0.10, $\eta_{p}^{2}=0.33.$ There was a significant interaction between image and negation, *F*(1, 7) = 1027.69, MSE = 0.27, *p* < 0.05, $\eta_{p}^{2}=0.99,$ revealing that greater adaptation was found for same image unaltered test stimuli than same image negated stimuli (mean difference = 5.31, *p* < 0.05) and different negated test stimuli than different unaltered stimuli (mean difference = 2.96, *p* > 0.05). There was also an interaction between negation and orientation, *F*(1, 7) = 68.50, MSE = 0.89, *p* < 0.05, $\eta_{p}^{2}=0.91.$ Simple effects showed that the effect of orientation was larger for unaltered stimuli (mean difference = 14.490, *p* < 0.05) than for negated stimuli (mean difference = 10.577, *p* < 0.05). Finally, there was a three-way interaction, *F*(1, 7) = 167.24, MSE = 1.12, *p* < 0.05, $\eta_{p}^{2}=0.96.$

### Upright photographic negated adaptor

A parallel analysis was run for when the adaptor was a negated image (Figure 3B). This revealed a significant effect of image, whereby greater adaptation was observed when the same image was used at adaptation and test than when a different image was used, *F*(1, 7) = 288.52, MSE = 14.95, *p* < 0.05, $\eta_{p}^{2}=0.98.$ There was also a significant effect of orientation, whereby greater adaptation was observed when the test stimuli were upright than when they were inverted, *F*(1, 7) = 350.06, MSE = 6.84, *p* < 0.05, $\eta_{p}^{2}=0.98.$ There was a significant interaction between image and orientation, *F*(1, 7) = 123.82, MSE = 1.88, *p* < 0.05, $\eta_{p}^{2}=0.95.$ Simple main effects showed that the magnitude of adaptation was stronger for negated images than unadjusted images when the same image was used as the adaptor as those that made up the test morph continua (mean difference = 2.63, *p* < 0.05), whereas the magnitude of adaptation was stronger for unadjusted images than negated images when a different image was used as the adaptor to that at test (mean difference = 2.79, *p* < 0.05). There was also an interaction between negation and orientation, *F*(1, 7) = 28.12, MSE = 1.35, *p* < 0.05, $\eta_{p}^{2}=0.80,$ which revealed itself in a greater magnitude of adaptation for negated upright stimuli than inverted stimuli (mean difference = 13.46, *p* < 0.05) which was greater than when the stimuli were unadjusted (mean difference = 10.69, *p* < 0.05).

### Inverted photographic positive adaptor

A further parallel analysis was run on the data when the adaptor was inverted (Figure 3C). This revealed a significant effect of image, whereby the same image produced greater adaptation than a different image, *F*(1, 7) = 115.93, MSE = 7.64, *p* < 0.05, $\eta_{p}^{2}=0.94.$ There was also a main effect of negation, whereby there was less adaptation for negated images than control images, *F*(1, 7) = 733.48, MSE = 1.83, *p* < 0.05, $\eta_{p}^{2}=0.99.$ Finally, there was a significant interaction between image and orientation, *F*(1, 7) = 18.82, MSE = 10.99, *p* < 0.05, $\eta_{p}^{2}=0.73,$ revealing itself through greater magnitude of adaptation for same upright images than same inverted images (mean difference = 1.95, *p* < 0.05) and different inverted images than different upright images (mean difference = 2.33, *p* < 0.05). No other effects were significant.

### Inverted photographic negated adaptor

A fourth analysis was run on the data for when the adaptor was both inverted and negated (Figure 3D). This revealed a significant effect of image, whereby greater adaptation was observed when the adaptation and test stimuli matched than when they were different, *F*(1, 7) = 373.41, MSE = 0.87, *p* < 0.05, $\eta_{p}^{2}=0.98.$ There was a significant effect of negation, whereby greater adaptation was observed when the test stimuli were not negated than when they were, *F*(1, 7) = 65.24, MSE = 0.54, *p* < 0.05, $\eta_{p}^{2}=0.90.$ There was also a main effect of orientation, *F*(1, 7) = 261.97, MSE = 11.42, *p* < 0.05, $\eta_{p}^{2}=0.97,$ whereby inverted test stimuli were less adapted to than upright test stimuli. There was an interaction between image and orientation, *F*(1, 7) = 24.68, MSE = 3.49, *p* < 0.05, revealing itself through a larger main effect of orientation when the test stimuli were different from the adaptor (mean difference = 16.96, *p* < 0.05) than when the test stimuli were the same as the adaptor (mean difference = 10.39, *p* < 0.05). Finally, there was an interaction between negation and orientation, *F*(1, 7) = 39.47, MSE = 0.69, *p* < 0.05, $\eta_{p}^{2}=0.85.$ Simple effects revealed that the main effect of orientation was greater for negated test stimuli (mean difference = 14.98, *p* < 0.05) than for unaltered test stimuli (mean difference = 12.38, *p* < 0.05).

## Experiment 1B – Unfamiliar Faces

All aspects of the method were identical to Experiment 1a, except that a different set of 32 participants were recruited and were tested on unfamiliar faces. The unfamiliar faces were matched for image quality to the famous faces, but were from the NimStim Face Stimulus Set (Tottenham et al., 2002) and had been previously rated as a similar level of attractiveness and distinctiveness as the famous faces in a pretest. They were matched and morphed in the same way as in Experiment 1a. The procedure contained an extra phase when the participants were introduced to the faces (prior to the baseline). Participants were shown each face identity (with either the letter T or G underneath) for 5 s five times. Then they were presented the faces 10 times without the letter and asked to identify the face (by pressing either T or G). Participants were given feedback. After these trials, the participants were given a further 10 trials without feedback. Accuracy was above 95% for all participants at this point. Following this, the procedure was identical to Experiment 1a.

## Results

**Figure 4:**
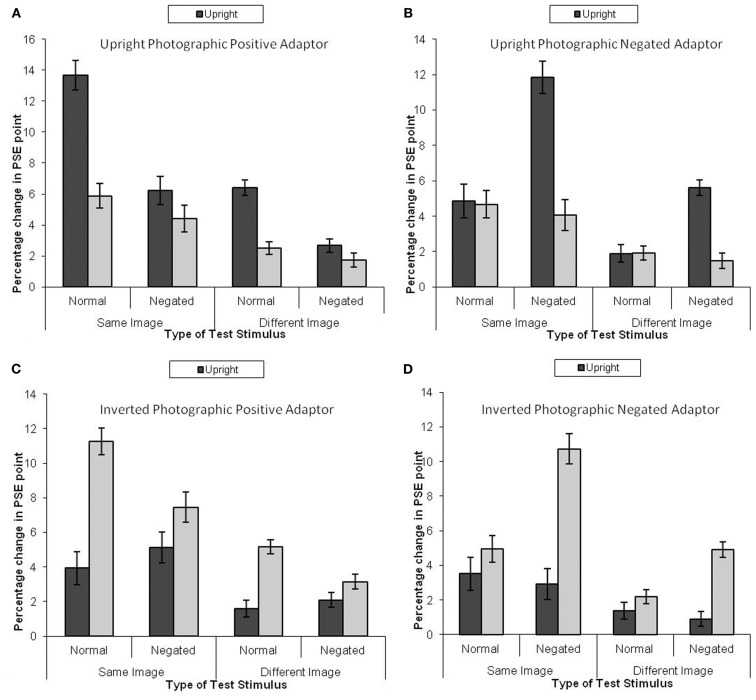
**Mean PSE (in identity strength needed to perceive the adapted identity) shift pre- to post-adaptation (as a measure of the magnitude of the aftereffect) for unfamiliar faces, when the adaptor is (A) upright and photographically positive, (B) upright and negated, (C) inverted and positive, and (D) inverted and negated**. Darker bars represent upright test stimuli, lighter bars represent inverted test stimuli. Error bars represent standard error.

The analysis protocol was identical for Experiments 1a and 1b, and the mean PSE shift is presented in Figure 4. This revealed a significant four-way interaction, *F*(3, 28) = 27.59, MSE = 0.45, *p* < 0.05, $\eta_{p}^{2}=0.75,$ similar to Experiment 1a. There main effect of adaptor type was not significant, *F*(3, 28) = 1.44, MSE = 18.10, *p* > 0.25, $\eta_{p}^{2}=0.13.$

### Upright photographic positive adaptor

Figure 4 indicates that greater adaptation occurred when the test stimuli were upright (Figure 4A). The data were subjected to a 2 × 2 × 2 within-subjects ANOVA. This revealed that greater adaptation occurred when the same image was used for both adaptation and test, *F*(1, 7) = 112.04, MSE = 2.54, *p* < 0.05, $\eta_{p}^{2}=0.94.$ Greater adaptation was observed for upright test stimuli than inverted test stimuli, *F*(1, 7) = 101.65, MSE = 2.06, *p* < 0.05, $\eta_{p}^{2}=0.94.$ Greater adaptation was observed for photographic positive stimuli than photographic negative stimuli, *F*(1, 7) = 77.14, MSE = 2.36, *p* < 0.05, $\eta_{p}^{2}=0.92.$ There was a significant interaction between image and negation, *F*(1, 7) = 114.88, MSE = 0.17, *p* < 0.05, $\eta_{p}^{2}=0.85,$ revealing that greater adaptation was found for same image unaltered test stimuli than same image negated stimuli (mean difference = 5.32, *p* < 0.05) and different negated test stimuli than different unaltered stimuli (mean difference = 3.11, *p* < 0.05). There was also an interaction between negation and orientation, *F*(1, 7) = 39.90, MSE = 2.01, *p* < 0.05. Simple effects showed that the effect of orientation was larger for unaltered stimuli (mean difference = 5.85, *p* < 0.05) than for negated stimuli (mean difference = 1.38, *p* < 0.05). Finally, there was a three-way interaction, *F*(1, 7) = 40.16, MSE = 0.23, *p* < 0.05, $\eta_{p}^{2}=0.85.$

### Upright photographic negate adaptor

A parallel analysis was run for when the adaptor was a negated image (see Figure 4B). This revealed a significant effect of image, whereby greater adaptation was observed when the same image was used at adaptation and test than when a different image was used, *F*(1, 7) = 76.62, MSE = 2.78, *p* < 0.05, $\eta_{p}^{2}=0.92.$ There was also a significant effect of orientation, whereby greater adaptation was observed when the test stimuli were upright, *F*(1, 7) = 57.58, MSE = 2.52, *p* < 0.05, $\eta_{p}^{2}=0.89.$ There were larger aftereffects in photographic positive images than negated images, *F*(1, 7) = 27.34, MSE = 3.41, *p* < 0.05, $\eta_{p}^{2}=0.80.$ There was a significant interaction between image and photographic negation, *F*(1, 7) = 14.02, MSE = 0.67, *p* < 0.05, $\eta_{p}^{2}=0.68.$ Simple effects showed that the magnitude of adaptation was stronger for negated images than unadjusted images when the same image was used as the adaptor as those that made up the test morph continua (mean difference = 3.18, *p* < 0.05), and when a different image was used as the adaptor to that at test (mean difference = 1.65, *p* < 0.05). There was also an interaction between negation and orientation, *F*(1, 7) = 63.71, MSE = 2.15, *p* < 0.05, $\eta_{p}^{2}=0.90,$ which revealed itself in a greater magnitude of adaptation for negated upright stimuli than inverted stimuli (mean difference = 5.94, *p* < 0.05) and no difference when the test stimuli were unadjusted (mean difference = 0.88, *p* = ns).

### Inverted photographic positive adaptor

A further parallel analysis was run on the data when the adaptor was inverted (Figure 4C). This revealed a significant effect of image, whereby the same image produced greater adaptation than a different image, *F*(1, 7) = 148.63, MSE = 1.68, *p* < 0.05, $\eta_{p}^{2}=0.96.$ There was also a main effect of negation, whereby there was less adaptation for negated images than control images, *F*(1, 7) = 6.19, MSE = 2.68, *p* < 0.05, $\eta_{p}^{2}=0.47.$ The main effect of orientation was significant, whereby there was more adaptation for inverted images than upright images, *F*(1, 7) = 83.80, MSE = 2.42, *p* < 0.05, $\eta_{p}^{2}=0.92.$ There was a significant interaction between photographic negation and orientation, *F*(1, 7) = 16.65, MSE = 3.38, *p* < 0.05, $\eta_{p}^{2}=0.70,$ in which the aftereffects were greater when the test images were inverted than upright when they were photographic positive (mean difference = 5.45, *p* < 0.05) but not when the test images were negated (mean difference = 1.69, ns). Finally, there was an interaction between orientation and image type, *F*(1, 7) = 117.64, MSE = 0.22, *p* < 0.05, $\eta_{p}^{2}=0.94,$ revealing itself through greater magnitude of adaptation for same inverted images than same upright images (mean difference = 4.83, *p* < 0.05) and different inverted images than different upright images (mean difference = 2.31, *p* < 0.05). No other effects were significant.

### Inverted photographic negate adaptor

A fourth analysis was run on the data for when the adaptor was both inverted and negated (Figure 4D). This revealed a significant effect of image, whereby greater adaptation was observed when the adaptation and test stimuli matched than when they were different, *F*(1, 7) = 97.87, MSE = 1.66, *p* < 0.05, $\eta_{p}^{2}=0.93.$ There was a significant effect of negation, whereby greater adaptation was observed when the test stimuli were negated than when they were not, *F*(1, 7) = 15.11, MSE = 3.67, *p* < 0.05, $\eta_{p}^{2}=0.68.$ There was also a main effect of orientation, *F*(1, 7) = 33.71, MSE = 5.85, *p* < 0.05, $\eta_{p}^{2}=0.83,$ whereby inverted test stimuli were more adapted to than upright test stimuli. There was an interaction between image and orientation, *F*(1, 7) = 29.60, MSE = 0.67, *p* < 0.05, $\eta_{p}^{2}=0.81,$ revealing itself through a larger main effect of orientation when the test stimuli were the same as the adaptor (mean difference = 4.62, *p* < 0.05) than when the test stimuli were the different to the adaptor (mean difference = 2.40, *p* < 0.05). There was an interaction between negation and orientation, *F*(1, 7) = 14.78, MSE = 6.23, *p* < 0.05, $\eta_{p}^{2}=0.68.$ Simple effects revealed that the main effect of orientation was greater for negated test stimuli (mean difference = 5.91, *p* < 0.05) than for unaltered test stimuli (mean difference = 1.11, ns). Finally, there was an interaction between photographic negation and image, *F*(1, 7) = 26.39, MSE = 0.33, *p* < 0.05, $\eta_{p}^{2}=0.79,$ whereby negated images had a larger aftereffect than positive images when the test images were the same as the adaptor (mean difference = 2.60, *p* < 0.05) than when the test images were different to the adaptor (mean difference = 1.12, ns).

## Summary

These results indicate that the interaction between type of processing, image degradation, and face-specific mechanisms (as indicated by the factors: orientation, photographic negation, and image-change) depends on what the adaptor is. This will be further discussed in the Section “General Discussion.” To address whether there are differences across familiarity, a five-way ANOVA combining Experiments 1a and 1b, thus containing the factors: familiarity (famous or unfamiliar faces); adaptor type; orientation (upright and inverted); negation (negated and normal); and image (same and different). Crucially, the five-way interaction was significant, *F*(3, 56) = 7.77, MSE = 1.76, *p* < 0.05, $\eta_{p}^{2}=0.29.$ Additionally, the main effect of familiarity was significant, *F*(1, 56) = 30.24, MSE = 13.78, *p* < 0.05, $\eta_{p}^{2}=0.81,$ in which aftereffects were significantly stronger for famous faces than unfamiliar faces (mean difference = 5.13). This suggests that the mechanisms behind adaptation in famous and unfamiliar faces are different. Further discussion of this is provided in the Section “General Discussion.”

## Experiment 2

A second experiment was conducted that aimed to examine how the FIAE is affected by other lower-level visual processing. Three different adaptors and three different test image manipulations were used. These compared the effects of high- and low-pass visual filtering on the FIAE in famous faces. Face identification is typically carried by spatial frequencies with a peak sensitivity between 8 and 13 cycles per degree (Näsänen, 1999), though higher spatial frequencies may be involved in early face identification (Halit et al., 2006). Based on this, identity aftereffects ought to be stronger for unaltered faces and low-pass faces. In addition to this, two further hypotheses (similar to Experiment 1) can be made regarding this study. Image-based adaptation may occur, whereby the greatest adaptation will be greater when the adaptor and test stimuli are matched, regardless of what the adaptor is. Alternatively, identity adaptation could occur, whereby aftereffects transfer across the image manipulations. Given that for identity recognition, a mismatch in spatial frequency of a single bandwidth from learning to test causes a recognition detriment of approximately 20% (Liu et al., 2000), we would expect that aftereffects should not transfer so readily across adaptors of one spatial frequency distribution to test stimuli of a different spatial frequency distribution.

## Method

### Participants and materials

Sixty Cardiff University students undertook this experiment as partial fulfillment of a course requirement. All participants had normal or corrected vision. All were White British nationals who were famous with the famous faces.

The unaltered image pair 1 and associated morphs from Experiment 1 was used here. Two additional sets were created that were either high- or low-pass filtered. This filtering was completed using MATLAB software. The original faces were put through a bandpass filter by multiplying together a low-pass and high-pass Butterworth filter using the equations presented in Collin et al. (2004). Subsequently, the images were inversely transformed into the spatial domain. The filtered faces had center frequencies of 7.08 (for the low-pass filtered faces) and 14.15 (for the high-pass filtered faces) cycles per face, with a bandwidth of 0.5 octaves. These center frequencies were chosen given that they are just outside the peak sensitivity bandwidth used in face identification (Näsänen, 1999).

### Design and procedure

A 3 × 3 mixed design was employed in which the type of adaptor was manipulated between subjects and the type of test stimuli was manipulated within-subjects. These were either unaltered, high- or low-pass filtered images. The experimental procedure was undertaken in the same way as Experiment 1.

## Results

**Figure 5:**
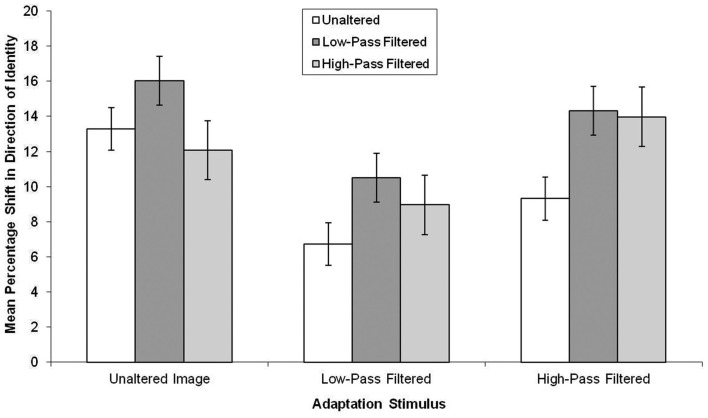
**Mean PSE (in identity strength needed to perceive the adapted identity) shift pre- to post-adaptation (as a measure of the magnitude of the aftereffect), for unaltered, low-pass, and high-pass filtered adaptation stimuli split by unaltered, low-pass, and high-pass filtered test stimuli for famous faces**. Error bars represent standard error.

The data, summarized in Figure 5, were subjected to a 3 × 3 mixed-subjects ANOVA. This revealed there was an effect of the test stimuli, *F*(2, 114) = 4.50, MSE = 49.47, *p* < 0.05, $\eta_{p}^{2}=0.07,$ in which aftereffects were smaller in unaltered test stimuli than low-pass filtered test stimuli (mean difference = 3.85, *p* < 0.05). There was also a main effect of adaptor type, *F*(2, 57) = 8.97, MSE = 46.35, *p* < 0.05, $\eta_{p}^{2}=0.24,$ in which, there was greater aftereffects following adaptation to the unaltered and high-pass filtered adaptors than the low-pass filtered faces (mean difference = 5.06, *p* < 0.05 and mean difference = 3.79, *p* < 0.05, respectively).

## Experiment 2B

Experiment 2b was conducted in the same way as Experiment 2a, except that the faces were unfamiliar (the same as those used in Experiment 1b. A different set of 60 participants were recruited.

## Results

**Figure 6:**
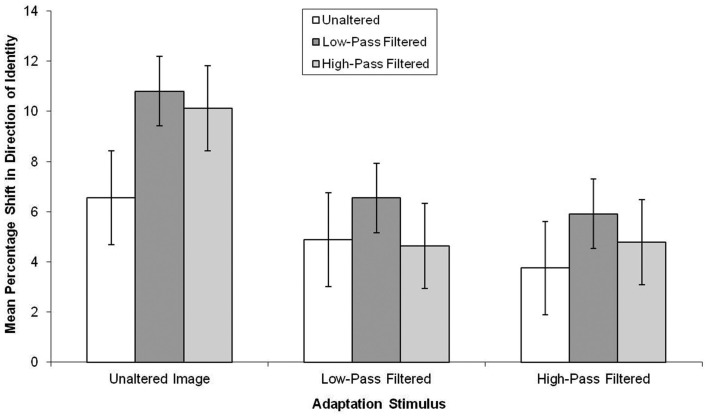
**Mean PSE (in identity strength needed to perceive the adapted identity) shift pre- to post-adaptation (as a measure of the magnitude of the aftereffect), for unaltered, low-pass, and high-pass filtered adaptation stimuli split by unaltered, low-pass, and high-pass filtered test stimuli for unfamiliar faces**. Error bars represent standard error.

The data, summarized in Figure 6, were subjected to a 3 × 3 mixed-subjects ANOVA. This revealed there was a marginal effect of the test stimuli, *F*(2, 114) = 2.78, MSE = 39.23, *p* = 0.07, $\eta_{p}^{2}=0.05,$ in which aftereffects were smaller in unaltered test stimuli than low-pass filtered test stimuli (mean difference = 2.69, *p* < 0.05). There was also a main effect of adaptor type, *F*(2, 57) = 3.27, MSE = 103.05, *p* < 0.05, $\eta_{p}^{2}=0.10,$ in which, there was greater aftereffects following adaptation to the unaltered than the high-pass and low-pass filtered faces, though not significantly (mean difference = 4.35, *p* = 0.07 and mean difference = 3.81, *p* = 13, respectively).

## Summary

Similar to Experiment 1, a comparison across famous and unfamiliar faces was conducted by inputting the data into a 2 × 3 × 3 mixed-subjects ANOVA with the factors: familiarity of the face, adaptor type, and type of test stimuli. This time, the three-way interaction was not significant, *F*(4, 228) = 1.17, MSE = 44.35, *p* = 0.32, $\eta_{p}^{2}=0.02.$ The main effect of familiarity was significant, *F*(1, 114) = 33.10, MSE = 74.70, *p* < 0.05, $\eta_{p}^{2}=0.23,$ in which aftereffects were greater following adaptation to famous faces (mean difference = 5.24). Taken together, these results indicate that image degradation affects the FIAE famous and unfamiliar faces in a similar manner. However, aftereffects are greater in famous faces than unfamiliar faces.

## General Discussion

Across all conditions, the magnitude of the aftereffect was largest when the adaptor and the test stimuli matched. In fact, when the adaptor and test stimuli matched, the aftereffect was twice that of when they did not match. This indicates that the FIAE is based, at least partially, on low-level mechanisms. Specifically, approximately half of the observed aftereffect is low-level. This type of aftereffect is the same across famous and unfamiliar faces as revealed by the lack of significance of the three-way interaction in Experiment 2. In addition, there were differences across the nature of the FIAEs for famous and unfamiliar faces as revealed by the five-way interaction in Experiment 1. The results for the unfamiliar faces directly replicate those found by Yamashita et al. (2005) when testing the FDAE. However, the results for famous faces are not consistent with these results (Experiment 1b).

Adaptation transfers across photographic negation and to a different image of the same face to a similar degree. Thus, photographic negation does not affect the FIAE in famous faces, possibly because it does not affect face-specific processing mechanisms. Identity can still be extracted quickly from a negated face, so the added error during encoding does not influence adaptation (see Figures 3C,D). Similarly, Experiment 2 demonstrated that low-level visual alterations to faces had similar effects on famous and unfamiliar faces, except that the aftereffects were typically larger in famous faces than unfamiliar faces. This is likely to be caused by more robust representations of famous faces (Ryu and Chaudhuri, 2006) and stronger aftereffects in famous faces than in unfamiliar faces (Carbon and Leder, 2005; Carbon and Ditye, 2012).

More interestingly, adaptation to an upright stimulus does not transfer to inverted test stimuli. This suggests that during the test phase, extracting identity from the inverted faces is unaffected by adaptation. This may be because it takes longer to recognize an inverted face (Valentine, 1988) and the response to the face is made before recognition is fully made (cf., the difference between a remember and a know response in the remember/know procedure).

The results are more intriguing when the adaptor is inverted. Here, the adaptation does transfer to upright stimuli (Figures 3B,D and 4B,D). However, the magnitude of adaptation depends on how different the test stimuli are from the adaptor. The magnitude of adaptation is smaller when there are more differences between the adaptor and the test stimuli. When the adaptor is inverted and negated (Figures 3D and 4D), the magnitude of adaptation does not depend on degree of difference between the adaptation stimuli and the test stimulus, since greater adaptation was noted for upright test stimuli.

These data are broadly consistent with those of Yamashita et al. (2005), in that the present study observed a transfer of adaptation from unaltered stimuli to negated images that was half that when the images matched. Yamashita et al. reported that this kind of transfer is small but still present. Here, we found that the effect was larger in famous faces than Yamashita et al. found and in unfamiliar faces. Perhaps, aftereffects in famous faces are more robust than in unfamiliar faces (Carbon and Leder, 2005, 2006) and more resistant to image manipulations.

These results are also somewhat different to those presented by Watson and Clifford (2006) in terms of the asymmetry of the adaptation effects transferring across upright and inverted faces. Specifically, here, we found that adaptation transfers more when the adaptor is inverted and the test stimuli are upright than vice versa. Watson and Clifford (2003, 2006) found the opposite asymmetry using unfamiliar faces. This highlights another difference in adaptation to famous and unfamiliar faces. Watson and Clifford (2003, 2006) explored aftereffects using different distortions to ours (gender-judgment and stretched faces). Thus, the differences in our results to Watson and Clifford may simply reflect different mechanisms in the FDAE compared to the FIAE. While this is possible, many authors suggest that the mechanisms for FDAE and FIAE are based on the shifting of a face prototype which suggests that the results ought to be comparable. Indeed, our results are consistent with those presented by Hole (2011) suggesting that familiarity is the critical variable here rather than methodological differences.

To explain why adaptation does not transfer from upright adaptation stimuli to inverted test stimuli could be based on the notion of expert face processing for upright faces. Since negation does not alter the manner of expert processing, this is plausible to explain the results when the adaptation stimuli are upright. However, this explanation fails to account for the successful transfer of adaptation from inverted adaptation stimuli to upright test stimuli. Evidence has been presented to suggest that aftereffects in inverted faces are based on low-level visual processing rather than face-specific mechanisms (Susilo et al., 2010) so the transfer may not be expected. However, Susilo et al. tested the FDAE in unfamiliar faces. Thus, the nature of identity adaptation is more complicated than based on visual or expert face-processing skills.

One plausible explanation for the transfer from inverted adaptation stimuli to upright test stimuli may be based upon how participants process an inverted face. Extracting identity from an inverted face takes longer than in an inverted face, however, it is still completed within 5 s (Valentine, 1988) which is the length of time the adaptor was on screen for in the present study. Thus, even an inverted face can cause identity adaptation. However, an inverted test stimulus will not be affected by adaptation possibly because the presentation is too brief to activate the expert face recognition system.

Another possibility is that with briefer presentation, an inverted face does not give as much semantic information as an upright face. Whereas, prolonged exposure of an inverted face provides enough time to access semantic information about that face and its identity. In this way, an inverted face as the adaptation stimulus may allow participants to actively think about the identity of the person, whereas the brief presentation during the test phase may not. Nevertheless, these results need to be confirmed using a larger stimulus set (see, e.g., Carbon and Ditye, 2012).

Experiment 2 demonstrated that aftereffects were observed more strongly in low-pass filtered faces. This is likely to be the result of the fact that identity is carried in higher spatial frequencies (Schyns et al., 2002) and during early face processing (Halit et al., 2006). Based on this, during the test phase, participants are making quick responses and this response may rely on early face coding. This would make aftereffects appear larger in low-pass filtered faces. Rather surprisingly, aftereffects were greater following adaptation to unaltered and high-pass filtered stimuli than to the low-pass filtered stimuli. A mismatch in spatial frequency for learning to test in a recognition paradigm typically causes recognition deficits of approximately 20% (Liu et al., 2000) but there was no consistent reduction in the aftereffect when spatial frequencies did not match in this aftereffect paradigm. Potentially, this may relate to how the faces are processed. Early face processing relies on higher spatial frequencies, but later processing is more dependent on lower spatial frequencies (Halit et al., 2006). The adaptor is on screen for 5 s and which means that the early face processing could be inhibited in favor of later face processing using spatial frequencies in the lower bands. Thus, if the faces are stored more with low spatial frequencies than high spatial frequencies, then aftereffects are likely to be larger. This explanation can only be made hesitantly and requires further testing to see if during the adaptation phase, whether high or low spatial frequencies are employed.

This study highlights differences in the representation between famous and unfamiliar faces as revealed by aftereffects. Aftereffects in unfamiliar faces are more likely to be based on non-face-specific visual mechanisms (cf., Ryu and Chaudhuri, 2006). The FIAE in unfamiliar faces may actually be a variant of the FDAE since they are tested in similar paradigms and in unfamiliar faces identity is not the same as it is in unfamiliar faces. Thus, aftereffects in unfamiliar faces are likely to be low-level and more viewpoint-dependent. The neuroanatomical locus for this is likely to be in the striate cortex and the fusiform gyrus (cf., Hole, 2011; Hurlbert, 2001). However, identity is represented elsewhere in the brain (Rotshtein et al., 2005; Gobbini and Haxby, 2007; Kriegeskorte et al., 2007) and aftereffects in famous faces are likely to involve these areas (Hills et al., 2010). Indeed, viewpoint-invariant aftereffects have been found to be located in more posterior regions such as the posterior cingulate cortex, and the anterior temporal lobes (e.g., Eger et al., 2005; Furl et al., 2007). Face detection is said to involve the fusiform gyrus, whereas identity extraction involves the anterior inferotemporal cortex (Kriegeskorte et al., 2007). This is consistent with the suggestion that famous faces recruit additional brain regions that are more anterior than the fusiform gyrus. Thus, adaptation in famous faces is likely to involve more brain regions than adaptation in unfamiliar faces and lead to more robust aftereffects. This description is of course speculative and further research is required to confirm these suggestions.

One caveat with the explanations provided thus far and with the study in general is that there are substantial representational differences in faces of different levels of familiarity including the recruitment of different brain regions (Taylor et al., 2009). The results presented here only show how the adaptation is different in famous and unfamiliar faces which is not necessarily a novel finding. Nevertheless, this study has developed methods for investigating how faces of different levels of familiarity are stored: this method could be used to further elucidate different processing streams for familiar and unfamiliar faces (cf., sex-contingent aftereffects, Little et al., 2005) which is often ignored in the aftereffects literature. Similarly, given that there is behavioral evidence from recognition paradigms that other-race faces are not processed using the expert face-processing system (Tanaka et al., 2004), this method could be used to establish how different the processing of other-race faces is: if there is transfer of aftereffects from own- to other-race faces, then this suggests they are processed using similar mechanisms. If there is no transfer then the mechanisms used to process faces is likely to be different. This paradigm, thus, has scope for exploring the representation of different classes of faces.

In conclusion, these data seem to suggest two important facets of the FIAE. Firstly, there is some image-based adaptation that is occurring. This is lower-level and may exist to allow for differences between stimuli to be better detected. This is based on the fact that stimuli that are matched at adaptation and at test produce stronger FIAEs than unmatched stimuli. Secondly, part of the FIAE is based on face-specific mechanisms, since the FIAE is based in part on expert processing. As such FIAEs represent a unique class of high-level shape aftereffect due to expert processing involved in face processing, possibly based on configural coding.

## Conflict of Interest Statement

The authors declare that the research was conducted in the absence of any commercial or financial relationships that could be construed as a potential conflict of interest.

## Footnotes

^1^1It must be noted that some authors have found that inverted faces are processed both featurally and configurally but to different degrees (see, e.g., Miellet et al., 2011) given that there is a linear and not a step change in the how rotation affects face perception (Edmonds and Lewis, 2007; van der Linde and Watson, 2010). This suggests that inverted faces are processed *more* featurally than upright faces and *less* configurally. In other words, inverted faces are processed in a much less expert and face-specific manner than upright faces.

^2^While a non-face class of stimuli could be employed to ensure that we are really testing face-specific mechanisms, it is generally accepted that inverted faces are a useful control since they match upright faces on all low-level visual characteristics, but do not recruit “face-specific” brain regions (Gauthier et al., 1999), nor have the same “face-specific” ERP (e.g., Eimer, 2000).

## References

[B1] Anderson N. D. Wilson H. R. (2005). The nature of synthetic face adaptation. Vision Res. 45, 1815–1828 10.1016/j.visres.2005.01.012 15797771

[B2] Benton C. P. Jennings S. J. Chatting D. J. (2006). Viewpoint dependence in adaptation to facial identity. Vision Res. 46, 3313–3325 10.1016/j.visres.2006.06.002 16844181

[B3] Burton A. Wilson S. Cowan M. Bruce V. (1999). Face recognition in poor-quality video: evidence from security surveillance. Psychol. Sci. 10, 243–248 10.1111/1467-9280.00144

[B4] Carbon C. C. Ditye T. (2012). Face adaptation effects show strong and long-lasting transfer from lab to more ecological contexts. Front. Psychol. 3:3 10.3389/fpsyg.2012.00003 22291676 PMC3264890

[B5] Carbon C. C. Leder H. (2005). Face adaptation: changing stable representations of familiar faces within minutes? Adv. Exp. Psychol. 1, 1–7

[B6] Carbon C. C. Leder H. (2006). The Mona Lisa effect: Is “our” Lisa fame or fake? Perception 35, 411–414 10.1068/p5452 16619955

[B7] Carbon C. C. Strobach T. Langton S. R. H. Harsányi G. Leder H. Kovács G. (2007). Adaptation effects of highly familiar faces: immediate and long lasting. Mem. Cognit. 35, 1966–1976 10.3758/BF03192929 18265612

[B8] Collin C. A. Liu C. H. Troje N. F. McMullen P. A. Chaudhuri A. (2004). Face recognition is affected by similarity in spatial frequency range to a greater degree than within-category object recognition. J. Exp. Psychol. Hum. Percept. Perform. 30, 975–987 10.1037/0096-1523.30.5.975 15462634

[B9] Diamond R. Carey S. (1986). Why faces, are, and are not special: an effect of expertise. J. Exp. Psychol. Gen. 115, 107–117 10.1037/0096-3445.115.2.107 2940312

[B10] Edmonds A. J. Lewis M. B. (2007). The effect of rotation on configural encoding in a face-matching task. Perception 36, 446–460 10.1068/p5530 17455758

[B11] Eger E. Schweinberger S. R. Dolan R. J. Henson R. N. (2005). Familiarity enhances invariance of face representations in human ventral visual cortex: fMRI evidence. Neuroimage 26, 1128–1139 10.1016/j.neuroimage.2005.03.010 15961049

[B12] Eimer M. (2000). Effects of face inversion on the structural encoding and recognition of face: evidence from event-related brain potentials. Cogn. Brain Res. 10, 145–158 10.1016/S0926-6410(00)00038-0 10978702

[B13] Ellis H. D. Shepherd J. W. Davies G. M. (1979). Identification of familiar and unfamiliar faces from internal and external features: some implications for theories of face recognition. Perception 8, 431–439 10.1068/p080721 503774

[B14] Ferster D. Miller K. D. (2000). Neural mechanisms of orientation selectivity in the visual cortex. Annu. Rev. Neurosci. 23, 441–471 10.1146/annurev.neuro.23.1.441 10845071

[B15] Furl N. van Rijsbergen N. J. Treves A. Dolan R. J. (2007). Face adaptation aftereffects reveal anterior medial temporal cortex role in high level category representation. Neuroimage 37, 300–310 10.1016/j.neuroimage.2007.04.057 17561416 PMC2706324

[B16] Galper R. E. (1970). Recognition of faces in photographic negative. Psychon. Sci. 19, 207–208

[B17] Gauthier I. Tarr M. J. Anderson A. W. Skudlarski P. Gore J. C. (1999). Activation of the middle fusiform ‘face area’ increases with expertise in recognizing novel objects. Nat. Neurosci. 2, 568–573 10.1038/9224 10448223

[B18] George N. Dolan R. J. Fink G. R. Baylis G. C. Russell C. Driver J. (1999). Contrast polarity and face recognition in the human fusiform gyrus. Nat. Neurosci. 2, 574–580 10.1038/9230 10448224

[B19] Gobbini M. I. Haxby J. V. (2007). Neural systems for recognition of familiar faces. Neuropsychologia 45, 32–41 10.1016/j.neuropsychologia.2006.04.015 16797608

[B20] Halit H. de Haan M. Schyns P. G. Johnson M. H. (2006). Is high-spatial frequency information used in the early stages of face detection? Brain Res. 1117, 154–161 10.1016/j.brainres.2006.07.059 16999942

[B21] Hills P. J. Elward R. L. Lewis M. B. (2008). Identity adaptation is mediated and moderated by visualisation abilities. Perception 37, 1241–1257 10.1068/p5834 18853559

[B22] Hills P. J. Elward R. L. Lewis M. B. (2010). Cross-modal face identity aftereffects and their relation to priming. J. Exp. Psychol. Hum. Percept. Perform. 36, 876–891 10.1037/a0018731 20695706

[B23] Hole G. (2011). Identity-specific face adaptation effects: evidence for abstractive face representations. Cognition 119, 216–228 10.1016/j.cognition.2011.01.011 21316651

[B24] Hurlbert A. (2001). Trading faces. Nat. Neurosci. 4, 3–5 10.1038/82877 11135632

[B25] Jiang F. Blanz V. O’Toole A. J. (2006). Probing the visual representation of faces with adaptation: a view from the other side of the mean. Psychol. Sci. 17, 493–500 10.1111/j.1467-9280.2006.01734.x 16771799

[B26] Jiang F. Blanz V. O’Toole A. J. (2007). The role of familiarity in three-dimensional view-transferability of face identity adaptation. Vision Res. 47, 525–531 10.1016/j.visres.2006.10.012 17207832

[B27] Jiang F. Blanz V. O’Toole A. J. (2009). Three-dimensional information in face representations revealed by identity aftereffects. Psychol. Sci. 20, 318–325 10.1111/j.1467-9280.2009.02285.x 19207696

[B28] Kemp R. Towell N. Pike G. (1997). When seeing should not be believing: photographs, credit cards and fraud. Appl. Cogn. Psychol. 11, 211–222 10.1002/(SICI)1099-0720(199706)11:3<211::AID-ACP430>3.3.CO;2-F

[B29] Kriegeskorte N. Formisano E. Sorger B. Goebel R. (2007). Individual faces elicit distinct response patterns in human anterior temporal cortex. PNAS 104, 20600–20605 10.1073/pnas.0705654104 18077383 PMC2154477

[B30] Leopold D. A. O’Toole A. J. Vetter T. Blanz V. (2001). Prototype-referenced shape encoding revealed by high-level aftereffects. Nat. Neurosci. 4, 89–94 10.1038/82947 11135650

[B31] Levitt H. (1971). Transformed up-down methods in psychoacoustics. J. Acoust. Soc. Am. 49, 467–477 10.1121/1.1912388 5541744

[B32] Little A. C. DeBruine L. M. Jones B. C. (2005). Sex-contingent face aftereffects suggest distinct neural populations code male and female faces. Proc. R. Soc. Biol. Sci. 272, 2283–2287 10.1098/rspb.2005.3220 PMC1560190 16191641

[B33] Little A. C. DeBruine L. M. Jones B. C. Waitt C. (2008). Category contingent aftereffects for faces of different races, ages and species. Cognition 106, 1537–1547 10.1016/j.cognition.2007.06.008 17707364

[B34] Liu C. H. Collin C. A. Rainville S. J. Chaudhuri A. (2000). The effects of spatial frequency overlap on face recognition. J. Exp. Psychol. Hum. Percept. Perform. 26, 956–979 10.1037/0096-1523.26.3.956 10884004

[B35] Maurer D. Le Grand R. Mondloch C. J. (2002). The many faces of configural processing. Trends Cogn. Sci. (Regul. Ed.) 6, 255–260 10.1016/S1364-6613(02)01903-4 12039607

[B36] Megreya A. M. Burton A. M. (2006). Unfamiliar faces aren’t faces: evidence from a matching task. Mem. Cognit. 34, 865–876 10.3758/BF03193433 17063917

[B37] Miellet S. Caldara R. Schyns P. G. (2011). Local Jekyll and global Hyde: the dual identity of face identification. Psychol. Sci. 22, 1518–1526 10.1177/0956797611424290 22075238

[B38] Näsänen R. (1999). Spatial frequency bandwidth used in the recognition of facial images. Vision Res. 39, 353–383 10.1016/S0042-6989(99)00096-6 10748918

[B39] Rotshtein P. Henson R. N. A. Treves A. Driver J. Dolan R. J. (2005). Morphing Marilyn into Maggie dissociates physical and identity representations in the brain. Nat. Neurosci. 8, 107–113 10.1038/nn1370 15592463

[B40] Russell R. Sinha P. Biederman I. Nederhouser M. (2006). Is pigmentation important for face recognition? Evidence from contrast negation. Perception 35, 749–759 10.1068/p5490 16836042

[B41] Ryu J. Chaudhuri A. (2006). Representations of familiar and unfamiliar faces as revealed by viewpoint-aftereffects. Vision Res. 46, 4059–4063 10.1016/j.visres.2006.07.018 16996559

[B42] Schyns P. G. Bonnar L. Gosselin F. (2002). Show me the features! Understanding recognition from the use of visual information. Psychol. Sci. 13, 402–409 10.1111/1467-9280.00472 12219805

[B43] Susilo T. McKone E. Edwards M. (2010). Solving the upside-down puzzle: why do upright and inverted face aftereffects look alike? J. Vis. 10, 1–16 10.1167/10.3.12 21149314

[B44] Suzuki S. (2001). Attention-dependent brief adaptation to contour orientation: a high-level aftereffect for convexity? Vision Res. 41, 3883–3902 10.1016/S0042-6989(01)00249-8 11738454

[B45] Suzuki S. (2003). Attentional selection of overlapped shapes: a study using brief aftereffects. Vision Res. 43, 549–561 10.1016/S0042-6989(02)00683-1 12595000

[B46] Tanaka J. W. Kiefer M. Bukach C. M. (2004). A holistic account of the own-race effect in face recognition: evidence from a cross-cultural study. Cognition 93, B1–B9 10.1016/j.cognition.2003.09.011 15110726

[B47] Taylor M. Arsalidou M. Bayless S. Morris D. Evans J. Barbeau E. (2009). Neural correlates of personally familiar faces: parents, partner and own faces. Hum. Brain Mapp. 30, 2008–2020 10.1002/hbm.20641 18726910 PMC6870744

[B48] Tong F. Nakayama K. (1999). Robust representations for faces: evidence from visual search. J. Exp. Psychol. Hum. Percept. Perform. 25, 1015–1035 10.1037//0096-1523.25.4.1016 10464943

[B49] Tottenham N. Borscheid A. Ellertsen K. Marcus D. J. Nelson C. A. (2002). “Categorization of facial expressions in children and adults: establishing a larger stimulus set.” in Poster presented at the Cognitive Neuroscience Society Annual Meeting in San Francisco, April 2002, San Francisco

[B50] Valentine T. (1988). Upside-down faces: a review of the effect of inversion upon face recognition. Br. J. Psychol. 79, 471–491 10.1111/j.2044-8295.1988.tb02747.x 3061544

[B51] Valentine T. (1991). A unified account of the effects of distinctiveness, inversion, and race in face recognition. Q. J. Exp. Psychol. A 43, 161–204 10.1080/14640749108400966 1866456

[B52] van der Linde I. Watson T. (2010). A combinatorial study of pose effects in unfamiliar face recognition. Vision Res. 50, 522–533 10.1016/j.visres.2009.12.012 20043938

[B53] Watson T. L. Clifford C. W. G. (2003). Pulling faces: an investigation of the face-distortion aftereffects. Perception 32, 1109–1116 10.1068/p5082 14651323

[B54] Watson T. L. Clifford C. W. G. (2006). Orientation dependence of the orientation-contingent face aftereffect. Vision Res. 46, 3422–3429 10.1016/j.visres.2006.03.026 16723149

[B55] Webster M. A. MacLin O. H. (1999). Figural aftereffects in the perception of faces. Psychon. Bull. Rev. 6, 647–653 10.3758/BF03212974 10682208

[B56] Yamashita J. A. Hardy J. L. De Valois K. K. Webster M. A. (2005). Stimulus selectivity of figural aftereffects for faces. J. Exp. Psychol. Hum. Percept. Perform. 31, 420–437 10.1037/0096-1523.31.3.420 15982123

[B57] Yin R. K. (1969). Looking at upside-down faces. J. Exp. Psychol. 81, 141–145 10.1037/h0027474

[B58] Zhao L. Chubb C. (2001). The size-tuning of the face-distortion aftereffect. Vision Res. 41, 2979–2994 10.1016/S0042-6989(01)00202-4 11704237

